# From Survival to Living: A Comprehensive Analysis of Fibula Graft Complications, Functional Outcomes, and Quality of Life Following Reconstruction for Malignant Bone Tumors

**DOI:** 10.3390/cancers18101548

**Published:** 2026-05-10

**Authors:** Beatrice Jung, Isabel Sperrhake, Saskia Sachsenmaier, Tilmann Busse, Eren Demir, Maria Christina Stefanescu, Constantin Doetsch, Sophie Zorn, Frank Traub

**Affiliations:** 1 Department of Orthopaedics and Trauma Surgery, University Medical Center Mainz, 55131 Mainz, Germanyfrank.traub@unimedizin-mainz.de (F.T.); 2 Department of Orthopaedics, University Hospital Tübingen, 72076 Tübingen, Germany

**Keywords:** malignant bone tumor, non-vascularized fibular graft, intercalary reconstruction, osteosynthesis failure, pseudarthrosis, functional outcome, quality of life

## Abstract

Background: Although survival rates for patients with malignant bone tumors have improved significantly, complications following tumor resection and limb-sparing reconstruction remain a major clinical challenge, particularly in young individuals. Intercalary resection often results in large bone defects, necessitating complex reconstructions. Fibula grafts offer biological advantages; however, their long-term outcomes, especially regarding mechanical complications and comprehensive patient-reported well-being, require further detailed exploration, particularly in cohorts utilizing non-vascularized grafts. Objective: This retrospective study evaluated the complication rates, bone hypertrophy, limb function, and quality of life following non-vascularized fibular graft reconstruction for malignant bone tumors in a single-center cohort. This study offers insights into long-term success and patient well-being, with a particular focus on correlations with systemic therapy and defect size, factors that remain insufficiently explored in the current literature. Methods: In this single-center retrospective study, twenty-four non-vascularized fibular grafts were used to reconstruct intercalary bone defects following malignant tumor resection. Complications were categorized using the Clavien–Dindo classification. Graft hypertrophy was evaluated according to the method described by Weiland and de Boer. Functional outcomes were assessed using the MSTSs and TESSs, while quality of life was measured using the SF-36 questionnaire. Notably, the cohort analyzed represents a relatively large single-center series focusing exclusively on the outcomes of non-vascularized fibular grafts. Results: Our findings revealed significant rates of mechanical complications, with osteosynthesis material failure occurring in 50.0% of cases, pseudarthrosis in 47.6%, and fractures of the fibular grafts in 38.1% of cases. Importantly, there were significant correlations between mechanical complications and systemic therapy (*p* = 0.017), as well as between defect size and fractures (*p* = 0.013), identifying critical risk factors. Despite these considerable complication rates, patients achieved satisfactory limb function (MSTS: 74 ± 17; TESS: 83 ± 15) and quality of life scores comparable to national norms, with notably higher mental health indices, highlighting their psychological resilience. Conclusions: Non-vascularized fibular graft reconstruction, despite high mechanical complication rates, significantly facilitates long-term functional recovery and psychological well-being. These findings emphasize the necessity of risk-adapted surgical strategies and long-term follow-up protocols to mitigate complications, optimize long-term function, and ultimately advance patient-centered care.

## Simple Summary

Patients with malignant bone tumors often need surgery to remove large parts of the affected bone. To restore the limb, doctors frequently use a piece of bone from the lower leg (fibula). Although this method has biological advantages, it can lead to complications such as fractures or problems with the implants. In this study, we examined the long-term outcomes of patients treated with this technique. We found that complications were com-mon; however, most patients regained good function and reported a quality of life similar to that of the general population. Interestingly, many patients also showed strong mental well-being. These results suggest that even when complications occur, patients can still achieve good long-term outcomes and maintain a good quality of life.

## 1. Introduction

Malignant bone tumors, although rare, represent a significant clinical burden and disproportionately affect children and young adults. Over the past three decades, patient survival has improved due to advances in multimodal therapy and refined surgical techniques that prioritize extremity preservation [1]. Currently, limb-sparing procedures are feasible in over 80% of cases, with no increase in local recurrence risk compared to amputation procedures [2,3]. However, the shift from amputation to limb salvage has introduced new challenges, including large post-resection bone defects, high mechanical stress zones, and long-term durability of reconstruction [4]. The focus has evolved from merely extending survival to achieving sustained physical function and a meaningful quality of life.

Among the available reconstructive techniques, fibular grafting remains a cornerstone, especially in younger patients [5,6]. Their anatomical properties, including dual vascular supply and ability to span large defects (up to 28 cm), make them particularly suitable for biological integration [7,8,9,10,11,12]. Depending on patient-specific factors, fibular grafts can be autologous, allogeneic, vascularized, or avascular. Each modality presents distinct trade-offs involving integration speed, mechanical strength, immune compatibility, and donor site morbidity. Understanding these trade-offs and the long-term performance of various graft modalities is crucial for achieving optimal patient outcomes.

Although previous studies broadly support the efficacy of fibular grafts, a notable limitation is that they often focus on select graft types, predominantly vascularized autografts, and report highly variable complication rates. Consequently, the existing literature critically lacks cohesive and granular data on specific long-term mechanical outcomes, such as pseudarthrosis, fractures, or implant failure, particularly across all graft modalities, including non-vascularized types. Furthermore, comprehensive assessments that holistically integrate radiologic evidence of biological integration, objective functional outcomes, and subjective patient-reported quality of life are notably scarce. This collective gap hinders a complete understanding of the long-term success and patient well-being following such complex reconstructions.

Therefore, to directly address these critical gaps, this retrospective study investigated a well-characterized cohort of 24 patients who underwent fibular graft reconstructions at a single center over a 10-year period, with a specific focus on non-vascularized fibular grafts, an approach that remains underrepresented in comprehensive long-term studies. To our knowledge, this is one of the largest case series of avascular fibular grafts used for the reconstruction of defects after resection of malignant bone tumors. Distinctively, our study provides an unprecedented comprehensive evaluation of long-term outcomes by meticulously analyzing mechanical complications, radiologic hypertrophy, objective limb function, and patient-reported quality of life—offering a multidimensional perspective. Moreover, correlations between systemic therapy, defect size, and complication profiles were rigorously explored, thereby informing crucial risk stratification and optimizing future reconstructive planning, an aspect often overlooked in broad outcome analyses.

This study aimed to provide a comprehensive assessment of long-term mechanical complications, radiologic graft integration, functional recovery, and psychosocial outcomes following non-vascularized fibular reconstruction. Particular emphasis was placed on correlations with defect size and systemic therapy as predictive factors. Ultimately, the goal is to bridge the gap between technical surgical success and holistic, patient-centered outcomes, reflecting the evolving paradigm in oncologic surgery that values not only survival but also sustained, meaningful quality of life.

## 2. Materials and Methods

After obtaining research ethics board approval (299/2020BO2), we reviewed our prospectively collected database to identify patients who underwent surgical resection of primary or secondary malignant bone tumors followed by reconstruction with non-vascularized fibular grafts between 2008 and 2018 at the University Hospital of Tübingen, Germany.

Inclusion criteria:

- Patients who underwent surgical resection of primary or secondary malignant bone tumors followed by reconstruction with non-vascularized fibular grafts between 2008 and 2018 at the University Hospital of Tübingen.
- Minimum follow-up of 12 months.
- Complete medical records and imaging studies available for review.

Exclusion criteria:

- Patients who underwent reconstruction with vascularized fibular grafts.
- Patients with benign bone tumors.
- Patients with incomplete medical records or imaging studies.
- Patients lost to follow-up before 12 months post-surgery.
- Patients who underwent amputation rather than limb salvage surgery.

Demographic, tumor-specific, and therapeutic data were extracted from prospectively collected records, including tumor histology, anatomical location, extent of bone resection, graft type, fixation method, and adjuvant treatment.

### 2.1. Surgical Technique

Reconstructions were performed across various anatomical regions: femur (*n* = 6), humerus (*n* = 6), tibia (*n* = 7), pelvis (*n* = 3), radius (*n* = 1), and clavicle (*n* = 1).

Fibular grafts were harvested using a posterolateral approach with preservation of the periosteum. To minimize peroneal nerve injury and maintain joint stability, the proximal fibular segment of 4–5 cm and the distal segment of 8–10 cm were preserved.

Fixation was achieved using plates in 21 cases. Based on the defect size, geometry, and anatomical site, reconstructions were performed using either single-strut (*n* = 14) or double-strut (*n* = 7) techniques. All tibial reconstructions utilized single-strut grafts, and femoral cases included four single- and two double-strut reconstructions. Single-plate fixation was used in cases involving the upper extremity or in patients weighing less than 40 kg.

### 2.2. Radiological and Functional Assessment

The biological activity at the graft–host interface was assessed using the hypertrophy index described by De Boer and Wood. A value >20% was defined as significant hypertrophy, 0–20% as biologically active without hypertrophy, and ≤0% as atrophy.

In pediatric patients, remodeling of the residual fibula was evaluated according to previously described criteria [12] and classified as complete, partial, or absent.

Functional outcomes were measured using the Musculoskeletal Tumor Society (MSTS) score. Additionally, patients completed the SF-36 and Toronto Extremity Salvage Score (TESS) questionnaires at the final follow-up. Physician-assessed MSTS scores were compared with patient self-assessments.

### 2.3. Follow-Up Protocol

Postoperative evaluations were conducted every 6–12 weeks until radiographic evidence of bone consolidation was confirmed. Standard radiographs were used to assess union at the graft-host junction, hypertrophy, tumor recurrence, and complications.

### 2.4. Complications and Classification

Complications, including infections, nonunion, fatigue fractures, local recurrences, and additional treatments (chemotherapy or radiotherapy), were recorded. Postoperative complications were classified according to the Clavien–Dindo classification.

### 2.5. Statistical Analysis

Statistical analyses were performed using SPSS v20.0 (SPSS Inc., Chicago, IL, USA). Descriptive statistics included the means, standard deviations, frequencies, and proportions. Age and time to consolidation were analyzed as continuous variables, and all other covariates were modeled categorically.

Comparisons of means between groups were performed using *t*-tests for continuous variables and chi-square tests for categorical variables. Post hoc testing was performed where appropriate. A multivariate analysis of factors influencing bone consolidation and functional outcomes was explored using a stepwise Cox proportional hazards regression, and the results should be interpreted with caution due to the limited sample size.

## 3. Results

### 3.1. Tumor Characteristics

The demographic and clinical characteristics of the included patients are listed in Table 1. A total of 24 tumors in 23 patients were treated with the fibula grafts. One boy with Li-Fraumeni syndrome initially presented with osteosarcoma in the femur and developed another osteosarcoma in the distal tibia on the same leg 4 years after completing the first treatment (No. 3 and 4 in Table 1). As the osteosarcomas differed histologically (one telangiectatic and one conventional osteoblastic osteosarcoma), they were not considered metastases.

The mean age at diagnosis was 17 ± 6.7 years for patients with osteosarcomas (29.2%), 14.1 ± 12.0 years for patients with Ewing sarcomas (33.3%), and 31 ± 10.2 years for patients with chondrosarcomas (20.8%). One patient had an adamantinoma (4.2%) and was 17.1 years old at diagnosis, and one patient with malignant fibrous histiocytoma (4.2%) was 76.2 years old at diagnosis. Most cases were classified as Enneking IIB (75%). In three cases (14.3%), the adjacent joint was also affected. Distribution of tumor localization: diaphyseal 54%, 20.55% metaphyseal and 20.8% were located at the metaphyseal/diaphyseal junction. Radiographic evidence of tumor regression was observed in 12/15 (80%) patients after neoadjuvant therapy. In two cases (13.3%), tumor growth was stable, and one case showed progression in the tibia. Nine patients (37.5%) did not receive neoadjuvant therapy due to diagnosis and treatment regimen, and in one case, information on tumor regression was missing. Surgical resection and neoadjuvant and adjuvant chemotherapy were administered in 15 (62.5%) patients with osteosarcoma or Ewing’s sarcoma. Adjuvant radiotherapy was additionally administered to one patient with Ewing’s sarcoma and one with mesenchymal chondrosarcoma. The mean defect length after surgical resection was 14 ± 5.7 cm (range: 5.5–28 cm).

### 3.2. Reconstruction

All 24 tumors underwent surgical resection of the affected bone and consecutive reconstruction of the defect with autologous or allogeneic fibula grafts. 21 grafts were fixed with locking plate osteosynthesis, one with K-wire osteosynthesis, and in two patients, the graft was applied in conjunction with pelvic spondylosyndesis using pedicle screws and rods. In 19 cases (79.2%), the fibula graft was harvested autologously from the ipsilateral side. Allogeneic transplantation was performed in five cases, all of which were donated by the patients’ fathers. In all selected cases, free non-vascularized transposition was performed.

### 3.3. Complications and Mortality

According to the Clavien–Dindo classification, only 4 of the 24 tumor reconstructions were free of complications. In 10 of the 24 cases, at least one additional surgical revision was required owing to complications.

The first complication occurred, on average, after 10.3 ± 6.1 months (95%CI: 7.3–13.3 months). Approximately 80% of the patients did not experience any complications 5 months after surgery. Approximately 30% of patients experienced complications after 10 months, ~20% after 15 months, and 12.5% after 20 months postoperatively. Upper and lower extremities accounted for 20.8% of cases in the upper extremity and 54.2% of the complications, respectively. Plate fractures occurred at an average of 30.3 ± 20.1 months (range, 13–59 months) postoperatively. Plate loosening occurred an average of 20.1 ± 13.8 months (range: 7–48 months) after the operation.

Complications occurred in 18 of 24 cases (75.0%), with 10 reconstructions (41.7%) requiring revision surgery. Material failure of plate osteosynthesis was observed in 12 of 24 cases (50.0%), predominantly in the lower extremities. Loosening was documented in 6 out of 21 cases (28.6%) at a mean of 20.1 ± 13.8 months, fractures in 3 patients (14.3%) at 30.3 ± 20.1 months, and a combination of both in another 3 cases (14.3%).

Specifically, for plate osteosynthesis, loosening occurred in six cases—two in the upper extremity and four in the lower extremity. One case of tibial plate fracture was recorded, while combined plate loosening and fracture were observed in three patients involving the femur and tibia. No significant correlation was found between the length of the bone defect and the lateral (*p* = 0.242) or medial (*p* = 0.283) plate length.

Reconstructions that received screw fixation in addition to plate osteosynthesis showed notably high complication rates. Screw loosening occurred in 6 patients (42.9%) at a mean of 14.4 ± 8.6 months (range: 1–27 months). Screw fractures were observed in three patients (21.5%) at 10.3 ± 6.0 months (range: 3–26 months), while a combination of loosening and fracture occurred in five cases (35.7%). Pseudarthrosis developed in five cases, including three hypertrophic and two hypotrophic forms, each associated with either graft fracture or hardware failure.

Patient age (*p* = 0.110), defect size (*p* = 0.443), and anatomical location (*p* = 0.514) did not significantly affect the occurrence of mechanical complications. However, a weak but statistically significant association was found between systemic therapy and mechanical complications (Fisher’s exact test: *p* = 0.017; Phi coefficient: *p* = 0.009).

A fracture of the fibula graft occurred in eight cases (38.1%), with the humerus (19.1%) being the most frequently affected site, followed by the tibia (9.5%) and femur (9.5%). The average time until the occurrence of a fibula graft fracture was 15.8 ± 7.9 months (range, 1–26 months) postoperatively. In three cases (37.5%), a second fracture occurred at 41.3 ± 13.9 months after the initial operation and 27.0 ± 18.3 months after the first fracture. The second fracture event was localized to a different site from the first fracture. No significant correlation was observed between anatomical localization and fracture occurrence (*p* = 0.257).

In 10 (47.6%) of 21 cases, pseudarthrosis of the fibula graft developed at the transition to the recipient bone, most frequently on the tibia (five cases), followed by the pelvis, humerus, clavicle, and femur. There was no significant correlation between the anatomical localization (*p* = 0.152), age (*p* = 0.217), systemic therapy (*p* = 0.562), or defect size (*p* = 0.231) and the occurrence of pseudarthrosis. Neurological complications upon removal of the fibula graft occurred in seven of 15 cases (46.7%). In 11 cases (52.4%), other complications occurred, including impaired wound healing, wound infections, and differences in the length of both extremities. The complication profile is presented in Table 2.

### 3.4. Bone Hypertrophy

The mean relative hypertrophy was calculated at 1, 3, and 5 years postoperatively. X-ray images of 20 cases were available for evaluation after 1 year, 17 cases after 3 years, and 10 cases after 5 years. According to the assessment of de Boer and Wood, hypertrophy after one year amounted to 21.2 ± 18.8% (range: 0–68%), 34.1 ± 27.7% (range: 0–90%) after three years, and 45.8 ± 35.1% (range: 4–100%) after five years. According to Weiland’s assessment, hypertrophy after one year amounted to 11.2 ± 3.7 points (range: 4–18 points), 12.2 ± 5.4 points (range: 0–18 points) after three years, and 14.6 ± 3.7 points (range: 8–18 points) after five years.

### 3.5. Functionality

At the end of the study, all 12 patients who underwent clinical examination demonstrated reduced mobility in the affected limbs. Specifically, light restrictions (less than 10°) were observed in seven cases, moderate restrictions (less than 20°) in two cases, and four patients exhibited no restrictions during this assessment. The mean MSTS score was 74 ± 17 points, with data available for 16 cases involving 15 patients. The overall mean TESS was 83 ± 15 points, with patients without complications achieving an average score of 83 ± 16 points, and those with complications attaining an average score of 86 ± 16 points.

### 3.6. Quality of Life

Fourteen patients completed the SF-36. The average score was 78 ± 11 points for all patients, 78 ± 11 points for patients with complications, and 83 ± 9 points for patients without complications. There was no significant correlation between the score and anatomical localization (*p* = 0.321).

Compared to the nationwide norm sample from the 1998 National Health Survey (physical component summary: 50.1 ± 9.9; mental component summary: 50.1 ± 10.4), patients in this study demonstrated slightly lower physical health scores (46.6 ± 9.7) and higher mental health scores (54.8 ± 5.8). This corresponds to an absolute difference of −3.5 ± 0.2 points for the physical component and +4.7 ± 4.6 points for the mental component, indicating better perceived mental health but slightly reduced physical function in the study cohort compared to the general population.

## 4. Discussion

This study offers a uniquely detailed, long-term evaluation of Fibula graft reconstruction outcomes in a homogeneous, single-institution cohort of 20 patients, distinguishing itself by its focus on non-vascularized grafts and comprehensive outcome assessment. With an average follow-up period exceeding 71 months, it comprehensively captures both early and delayed postoperative complications and late-stage biological remodeling [12,13,14,15,16,17,18]. Notably, the complication rates observed in our cohort—50.0% for osteosynthesis failure, 47.6% for pseudarthrosis, and 38.1% for graft fractures—were higher than those reported in some prior studies [12,14,16,17,18,19]. For instance, Frisoni et al. noted osteosynthesis failure in 22% of cases, whereas Schuh et al. noted it in only 7.6% of cases [17]. However, a crucial distinction lies in the composition of these previous cohorts, which often incorporated vascularized grafts known for superior remodeling and earlier hypertrophy and frequently employed narrower definitions of mechanical failure [12,15,16,17,18]. Consequently, the higher complication rates reported herein are highly reflective of our consistent use of non-vascularized fibula grafts and a more expansive definition of mechanical failure—encompassing screw loosening and component-specific events—thereby critically highlighting the inherent complexity of achieving long-term stability in large avascular reconstructions [12,15,16,17,18,19]. Despite these significant mechanical challenges, our study suggests that patients achieved good functional outcomes (MSTS 74 ± 17; TESS 83 ± 15) and exhibited strong QoL metrics, including superior mental health compared with national norms. These striking outcomes profoundly reinforce our central theme that successful limb salvage transcends mere technical reconstruction and contributes meaningfully to patients’ lived experiences and psychological well-being, even in the face of complications. A significant correlation was observed between systemic therapy and osteosynthesis failure (*p* = 0.017). This finding suggests that chemotherapy-induced suppression of osteogenesis and impaired bone remodeling may contribute to the early mechanical instability. Therefore, these results emphasize the importance of heightened surveillance and possibly altered fixation strategies in patients undergoing systemic therapy. Furthermore, a moderate correlation between defect length and graft fracture was identified (*p* = 0.013), with a median fracture-associated defect length of 14.7 cm. Specifically, defects ≥14.7 cm demonstrated a higher risk of fracture, particularly when reconstructed with a single strut [17,20]. These observations are consistent with prior reports by Lenze et al. [20] and Frisoni et al. [17]., who similarly noted an increased fracture risk with defects exceeding 12–17 cm. Consequently, double strut configurations or vascularized grafts may be better suited for these inherently larger and more mechanically demanding reconstructions [2,13,21].

No significant correlation was found between complication rates and age, defect location, or defect size (except for fractures). This aligns with Hilven et al.’s findings [19], but contrasts with studies such as Frisoni et al. [17]., which reported higher failure rates in patients > 18 years. Our younger cohort, concentrated in the second decade of life, may explain this variation.

Despite the high incidence of mechanical complications, radiographic assessments consistently show progressive and robust hypertrophy over time. This finding indicates that even non-vascularized grafts possess significant long-term remodeling capacity, achieving over 45% hypertrophy at five years postoperatively. Thus, this underscores the profound biological potential of fibula grafts and highlights the critical role of achieving and maintaining mechanical stability in the early postoperative period to facilitate such successful remodeling. Correspondingly, patients demonstrated impressive functional recovery, with no significant difference in TESSs between those who experienced complications and those who did not. This compelling evidence strongly suggests that timely and effective surgical revision, when required, can effectively mitigate the functional consequences of the initial mechanical failure and preserve long-term limb function.

Perhaps the most striking outcome was that patients reported better mental health than the general population, despite a history of limb-threatening malignancies and multiple surgeries. This highlights the profound psychological value of limb salvage and supports the evolving philosophy in sarcoma surgery, emphasizing not just survival but also living well. These findings reaffirm that biological reconstruction, even with its inherent risks, can deliver long-term satisfaction and resilience, particularly in young and motivated patient populations. Several alternative strategies to use fibula grafts have proven viable for reconstructing diaphyseal bone defects following tumor resection. Endoprosthetic reconstruction offers immediate mechanical stability and early mobilization, which is especially useful for elderly patients or those with limited life expectancy. Modular or custom intercalary endoprostheses yield high functional outcomes, although they carry the risks of aseptic loosening and mechanical failure over time [22,23]. In biological reconstruction, the Masquelet-induced membrane technique represents a promising two-stage approach that fosters graft incorporation through an osteoinductive membrane. Recent innovations, such as combining this technique with vascularized fibula or allografts (“Capasquelet” technique), have shown accelerated union and early weight-bearing, especially in complex or infected cases [24]. Bone transport via the Ilizarov technique enables biological regeneration of long defects without grafts, offering excellent outcomes in infection-prone settings or when donor material is limited. Despite its prolonged treatment duration, it yields high union and functional outcomes [25,26]. The choice of technique should be tailored to tumor location, patient age, infection status, and life expectancy to optimize onco-logic and functional outcomes.

### Limitations

While providing valuable insights, this study is subject to several limitations inherent to its design and scope of the study. First, the relatively small cohort size (*n* = 20) inherently limits the statistical power, particularly for robust subgroup comparisons, and may restrict the generalizability of our findings to a broader patient population. Second, as a retrospective cohort study, it is susceptible to potential selection and information bias. For instance, comprehensive data on all potential confounding factors, such as specific chemotherapy regimens or rehabilitation protocols, may not have been uniformly documented. Third, the absence of an internal control group, such as a direct comparison between vascularized and nonvascularized fibula grafts, restricts our ability to draw definitive conclusions regarding the superior efficacy or complication profiles of different graft types. This is particularly relevant given the observed higher complication rates in our predominantly non-vascularized cohort. Furthermore, although all surgeries were performed at a single center, minimizing protocol variability, the use of heterogeneous internal fixation techniques (e.g., single versus double strut, K-wire, and spinal stabilization) introduced a degree of variability that could influence mechanical outcomes. Although this reflects real-world clinical practice, it makes it challenging to isolate the impact of specific fixation methods. Finally, being a single-center study, the results may reflect specific institutional practices and patient demographics, potentially limiting external validity. Despite these recognized constraints, the granular and long-term insights derived from this well-characterized dataset offer rare and valuable contributions to the understanding of outcomes in this challenging surgical domain. However, these limitations also serve to inform the design of future, more robust investigations.

## 5. Clinical Implications and Future Directions

The findings from this study provide several crucial and actionable clinical implications for optimizing fibula graft reconstruction in patients with malignant bone tumors. Our identified correlations strongly suggest that patients undergoing systemic therapy or those requiring reconstruction for larger bone defects (specifically, a median fracture-associated defect length of 14.7 cm, indicating a threshold of >12–14 cm) should be designated as high-risk for mechanical complications, including osteosynthesis failure and graft fracture. Therefore, for these high-risk individuals, surgeons should consider more robust reconstructive strategies, such as the use of double-strut constructs or, where feasible, vascularized grafts, to enhance early mechanical stability. Despite the high complication rates, the sustained functional and psychological well-being observed underscores the importance of close postoperative monitoring and a low threshold for proactive and timely surgical revision. Early intervention for mechanical failures can effectively mitigate long-term functional deficits, ensuring that the benefits of limb salvage are achieved. Our results highlight the profound psychosocial benefits of limb salvage, even in the presence of physical challenges in the patient. Consequently, the routine incorporation of patient-reported outcome measures (PROMs), such as the SF-36 and TESS, should become a standard component of follow-up protocols for sarcoma reconstruction. This holistic assessment provides a more accurate and patient-centered evaluation of treatment success beyond mere oncological control or limb-preservation. Looking ahead, these insights pave the way for critical future research. Prospective comparative studies are warranted, specifically multicenter studies with larger cohorts, to validate our findings and directly compare the long-term outcomes, complication profiles, and functional results of vascularized versus non-vascularized fibula grafts. Such studies can provide definitive guidance for graft selection. Additionally, further research integrating advanced biomechanical modeling could elucidate the precise mechanical stresses on different graft configurations and fixation methods, especially in the context of large defects and systemic therapy, to inform optimized implant designs and surgical techniques. Finally, deeper longitudinal psychosocial evaluations are needed to explore the evolution of psychological well-being in this patient population and identify specific coping mechanisms or interventions that contribute to the observed resilience. By pursuing these directions, the orthopedic oncology community can continue to refine reconstructive strategies, minimize morbidity, and ultimately enhance the quality of life of patients undergoing limb salvage procedures.

## 6. Conclusions

This retrospective study provides a detailed and unique long-term evaluation of fibula graft reconstruction in a specific cohort of patients with malignant bone tumors, directly addressing gaps in the literature regarding the comprehensive outcomes of non-vascularized constructs. We revealed a notably high incidence of mechanical complications, most significantly osteosynthesis failure, pseudarthrosis, and graft fracture, which were particularly prevalent in the context of non-vascularized, single-strut reconstructions and larger bone defects. Crucially, however, despite these significant mechanical challenges, patients achieved remarkable long-term functional recovery and demonstrated consistently high levels of mental well-being, underscoring both the inherent durability and profound psychosocial benefits of limb salvage reconstruction. Therefore, our findings strongly reinforce the principle that mechanical failure, when effectively managed through timely revision and comprehensive follow-up, does not necessarily preclude meaningful long-term functional and psychological outcomes. Importantly, this study identified critical, actionable risk factors—namely, systemic therapy and increased defect size—that can directly guide preoperative planning, patient selection, and the strategic choice of reconstructive technique to mitigate potential complications. Furthermore, the observed progressive hypertrophy in non-vascularized grafts provides compelling evidence of the robust biological potential of these constructs, contingent on achieving adequate early mechanical stability. Overall, this study underscores the clinical value of integrating granular complication analysis with objective functional performance and subjective patient-reported outcomes into the holistic evaluation of limb salvage procedures. This firmly supports a paradigm shift in oncologic orthopedics, moving beyond the sole focus on survival to prioritizing sustained, high-quality living for patients facing complex musculoskeletal tumors. Management of diaphyseal bone defects following malignant tumor resection presents a multifaceted challenge in orthopedic oncology, necessitating careful selection of reconstructive strategies tailored to patient-specific factors. Vascularized fibula grafts (VFGs) have long been a mainstay of treatment due to their intrinsic osteogenic and remodeling capabilities. Previous studies demonstrated excellent long-term functional outcomes and survival of vascularized fibula grafts in humeral and tibial reconstructions, although early mechanical complications such as fractures and nonunion remain common [14,15,19,27]. Non-vascularized fibula grafts (NVFGs) may also provide satisfactory union and functional outcomes in selected patients [12]. Biological reconstruction options continue to expand, with composite grafts integrating devitalized tumor bone or allografts with VFGs, providing both structural integrity and biological potential [28]. Ogura et al. [15] reported high union rates and MSTS scores (~81%) using such techniques, albeit with risks of infection and implant failure. Pediatric studies have further validated the long-term viability of vascularized fibula grafts, reinforcing their role in younger patient populations [29]. New techniques are emerging as promising alternatives. The Masquelet-induced membrane technique has shown substantial utility in the management of infected or complex cases. This two-stage method, particularly in its hybrid form with vascularized grafts, fosters accelerated graft incorporation and early mobilization [24,30]. Likewise, the Ilizarov bone transport technique offers biologically regenerative potential without requiring grafts, which is especially beneficial in cases of infection or extensive bone loss. Despite its pro-longed treatment duration, it yields high union and functional outcomes [25]. Endoprosthetic reconstruction provides an alternative mechanical solution, especially in elderly patients or those with low survival rates. They allow immediate stabilization and function, although they are prone to long-term mechanical issues such as loosening [22,23]. This diversity of options highlights the need for individualized planning based on tumor characteristics, systemic therapy, age, and anticipated functional demands. In this context, the current study provides several critical insights. Despite the high complication rates associated with NVFGs, especially in large defects and post-chemotherapy patients, favorable long-term outcomes in function (MSTS 74 ± 17; TESS 83 ± 15) and quality of life were observed. Complications such as osteosynthesis failure (50.0%), pseudarthrosis (47.6%), and fractures (38.1%) were significantly associated with systemic therapy and larger defects (>14.7 cm), consistent with the findings of Frisoni and Lenze. Importantly, even non-vascularized grafts exhibited substantial hypertrophy (>45% at 5 years), emphasizing their latent biological capacity when mechanical integrity is sustained. Clinically, this evidence advocates for proactive surveillance, robust fixation (e.g., double-strut or hybrid constructs), and early revision strategies in high-risk patients. Furthermore, the observed psychological resilience in this cohort underscores the holistic value of limb salvage beyond structural repair, reaffirming the shift in sarcoma care from survival to well-being. These findings emphasize the pressing need for multicenter prospective trials comparing graft types, standardizing fixation strategies, and integrating biomechanical and psychosocial metrics to refine the future of biological reconstruction.

## Figures and Tables

**Table 1:** Demographic and clinical patient characteristics.

No.	Sex	Age	Diagnosis	Location	Stage	Defect (cm)	Chemo-Therapy	Radio-Therapy
1	female	17	osteosarcoma	humerus	III	24	neo + adj	-
2	male	6	Ewing	tibia	IIB	7	neo + adj	-
3	male	6	osteosarcoma	femur	IIB	16	neo + adj	-
4	male	11	osteosarcoma	tibia	IIB	10	neo + adj	-
5	female	33	chondrosarcoma	humerus	IIB	10	adj	adj
6	male	2	Ewing	femur	IIB	17	neo + adj	-
7	male	49	unknown	clavicle	III	12	-	-
8	male	9	Ewing	pelvis	IIB	6	neo + adj	-
9	female	22	osteosarcoma	tibia	IIB	16	neo + adj	-
10	male	24	osteosarcoma	tibia	IIB	17	adj	-
11	male	17	osteosarcoma	femur	IIB	18	neo + adj	-
12	male	22	Ewing	humerus	IIB	18	neo + adj	-
13	male	17	adamantinoma	tibia	IB	28	-	-
14	male	14	Ewing	tibia	IIB	15	neo + adj	-
15	female	76	MFH	humerus	-	11	-	-
16	female	53	sinus tumor	humerus	III	16	-	-
17	female	23	chondrosarcoma	pelvis	IIB	5	-	-
18	female	5	Ewing	femur	IIB	9	neo + adj	-
19	male	39	Ewing	tibia	IIB	15	neo + adj	adj
20	male	19	chondrosarcoma	radius	IIB	8	-	-
21	female	37	chondrosarcoma	femur	IA	8	-	-
22	female	56	osteosarcoma	pelvis	IIB	12	-	-
23	male	16	Ewing	humerus	IIB	14	-	-
24	female	44	chondrosarcoma	femur	IIB	22	-	-

**Table 2:** Complications and outcomes following fibular graft reconstruction.

Complication	*n* (%)	Time to Event (Months)
Any complication	18 (75.0)	10.3 ± 6.1
Revision surgery	10 (41.7)	—
Implant-related complications		
Plate loosening	6 (28.6)	20.1 ± 13.8
Plate fracture	3 (14.3)	30.3 ± 20.1
Screw loosening	6 (42.9)	14.4 ± 8.6
Screw fracture	3 (21.5)	10.3 ± 6.0
Combined screw failure	5 (35.7)	—
Biological complications		
Pseudarthrosis	10 (47.6)	—
Graft fracture	8 (38.1)	15.8 ± 7.9
Other complications		
Neurological complications *	7 (46.7)	—
Wound complications	11 (52.4)	—

* Revision surgery was performed in 10 cases (41.7%); *—*: not applicable.

## Data Availability

The data presented in this study are available on reasonable request from the corresponding author.
